# Electromagnetically Coupled Resonant Face‐to‐Face Double‐Layer Metamaterial for Highly Sensitive THz Impedance Spectroscopy

**DOI:** 10.1002/advs.202504331

**Published:** 2025-08-26

**Authors:** Rudrarup Sengupta, Heena Khand, Gabby Sarusi

**Affiliations:** ^1^ Department of Photonics and Electro‐Optics Engineering School of Electrical and Computer Engineering Ben‐Gurion University of the Negev Beer Sheva 8410501 Israel

**Keywords:** ELC THz metamaterial sensor, high‐Q resonance, optimal electromagnetic coupling, sensitivity enhancement, spectral shift at THz, THz spectroscopy

## Abstract

A new electromagnetic coupling mechanism is introduced between two passive terahertz (THz) electric‐LC resonator metasurfaces, aimed at maximising THz metamaterial impedance spectroscopy sensitivity, translated into resonance spectral red‐shift (Δ*F*). When two resonant metasurfaces are brought into a face‐to‐face proximity and exposed to THz radiation, a resonant optical cavity is generated, and an electromagnetic coupling of THz radiation occurs from both metasurfaces. This coupling triggers the enhancement of plasmonic interaction between the incident THz radiation and the metasurfaces. It is found out that an optimal distance between the metasurfaces is a critical parameter for achieving an enhanced coupling of the electrical fields. Once optimally coupled, the double‐layer metamaterial becomes an integrated sensor for THz impedance spectroscopy, exhibiting an enhanced resonance with a high Q‐factor of 549, without any major requirements for alignment of the two metasurfaces. The double‐layer metamaterial sensor achieves a high dielectric sensitivity of 2300 GHz RIU^−1^ that can detect a variety of inorganic and organic nanoparticles, as well as sugar in ultra‐low concentrations. As an example, precise blood sugar tracking is demonstrated defining clear distinctions between hypoglycaemia, normal, borderline high, and hyperglycaemia, all based on Δ*F*. This novel sensor architecture has potential applications in high‐sensitivity biosensing and ultra‐low‐concentration dielectric detection.

## Graphical abstract

A unique electromagnetic coupling between two ELC resonating metamaterial sensors in a THz optical cavity in face‐to‐face configuration, is copiously stable for any misalignment, and enhances resonant q‐factor to 549, dielectric sensitivity to 2300 GHz RIU^−1^; the highest to date. This sensor can detect any nanoparticle, sugar, biomolecules, etc., in ultra‐low concentrations with 23 times higher sensitivity compared to any standard metamaterial.

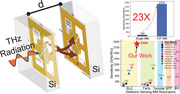

## 1 Introduction

Terahertz (THz) impedance spectroscopy using electric‐inductive‐capacitive (ELC) resonant metamaterial (MM) sensors^[^ ^1^, ^2^, ^3^, ^4^, ^5^, ^6^, ^7^, ^8^ ^]^ are progressively employed to detect various biomolecules and nanoparticles.^[^ ^9^, ^10^, ^11^, ^12^, ^13^, ^14^, ^15^, ^16^, ^17^, ^18^ ^]^When these particles, acting as dielectrics, are spread in the active capacitive gap (cap‐gap) of a metasurface with a 2D array of LC micro antennas, the original resonance frequency of the metamaterial is red‐shifted (Δ*F*).^[^ ^19^, ^20^, ^21^ ^]^ The extent of this shift indicates the sensor's dielectric sensitivity. Δ*F* is instrumental for particle sensing applications like identifying the presence and concentration of bacteria, viruses, and various inorganic nanoparticles on the metasurface.^[^ ^20^, ^22^, ^23^, ^24^, ^25^, ^26^, ^27^, ^28^, ^29^ ^]^ However, detecting particles at ultra‐low concentrations remains challenging due to the low dielectric sensitivity of most of the current ELC resonant metamaterials.^[^ ^19^, ^20^, ^21^, ^22^, ^23^, ^24^, ^30^, ^31^ ^]^

Enhancing dielectric sensitivity in ELC resonant metamaterials on conventional silicon (Si) substrates is challenging due to high permittivity and significant Fabry‐Pérot (FP) oscillations resulting in high absorption.^[^ ^19^, ^32^ ^]^ Popular methods for sensitivity enhancement, like using ultra‐thin non‐Si polymer substrates^[^ ^33^, ^34^, ^35^, ^36^ ^]^ and incorporating asymmetry structures to ignite dark modes^[^ ^37^, ^38^, ^39^, ^40^ ^]^ come with their own drawbacks of CMOS incompatibility and polarization dependency. In our previous works, we revealed FP oscillations‐MM resonance coupling physics, which limited the spectral shift (Δ*F*).^[^ ^19^ ^]^ We minimized the coupling by two methods, thinning down the substrate^[^ ^19^ ^]^ or attaching a back plate (or meta‐plate), resulting in eightfold increase in dielectric sensitivity.^[^ ^32^ ^]^ To date, the highest recorded sensitivity for an ELC resonator is 1050 GHz RIU^−1^.^[^ ^21^ ^]^ A dramatic improvement in dielectric sensitivity with a metamaterial sensor exhibiting high resonance quality is needed to detect a variety of inorganic and organic particles at ultra‐low concentrations.

This work explores a new concept of enhancing the electric field on the metasurfaces by optimal electromagnetic coupling between two metasurfaces, to maximize dielectric sensitivity. We have brought two ELC resonant metamaterial dies in face‐to‐face (F2F) configuration, at a proximity, creating an optical cavity, where the electric field from one metasurface is coupled with the other metasurface, at resonance. This resonant electromagnetic coupling between two metasurfaces triggers an enhanced plasmonic interaction between the incident THz radiation from the spectrometer and the resonant metasurfaces, resulting in a highly enhanced resonance quality factor along with very high spectral response in terms of Δ*F*, with a large dynamic range. We detected glucose with concentration levels starting form 20 mg/dl up to 150 mg/dl with a margin of 10 mg/dl, protein (Bovine Serum Albumin – BSA) from very low to high concentration, salts, and nanoparticles (ZnO and PbN), each with definitive range of Δ*F*, for each category of dielectrics. By utilizing this resonant electromagnetic coupling, in this work, we have recorded a high dielectric sensitivity of 2300 GHz/RIU.

## 2 Results and Discussion

### 2.1 Simulating the Distance for Optimal Electromagnetic Coupling of the Two Metasurfaces

**Figure 1:**
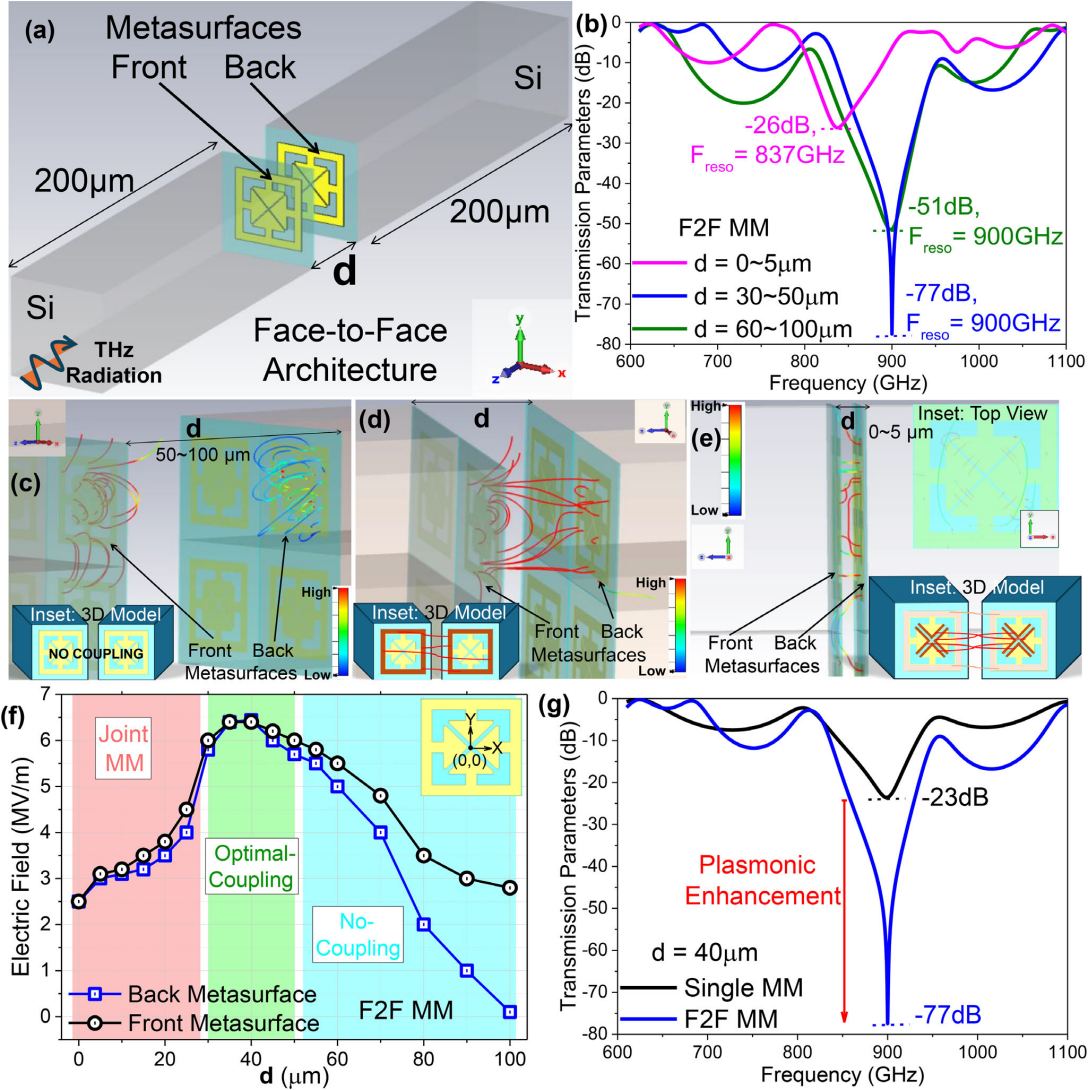
a) A 3D model of our double‐layer metamaterial sensor in face‐to‐face configuration (F2F) is shown with separation distance *d*. b) Simulated transmission parameters showing variation of resonance frequency (F_reso_) and transmission depths at resonance with varying *d*. Electric field streamlines at resonance are shown for the front and back metasurface, for c) *d*  =  60 − 100 µm, d) *d*  =  30 − 100 µm, and e) *d*  =  0 − 5 µm with the insets in d,e) explaining the method of electric field coupling. f) Simulated electric field at resonance is plotted at varying *d* probed at the center of the active area, showing three zones of coupling, joint MM, optimal‐coupling, and no‐coupling. g) Transmission parameters showing enhancement of plasmonic interaction, by a drastic increase of resonance depth, of our F2F MM, in optimal coupling distance of 40 µm.

**Figure** **1**a shows a 3D model of the face‐to‐face (F2F) double‐layer metamaterial sensor, consisting of two Si dies with 0.5 µm capacitive‐gap arrowhead metasurfaces (periodic structure in 2D array format) with a resonance frequency of 900 GHz.^[^ ^19^, ^32^ ^]^ They are placed in F2F configuration at different distances (*d*) to assess the coupling between the two metasurfaces and determine the optimal distance. Using CST 3D electromagnetic simulation (Figure 1b), we simulate THz transmission spectra where two metasurfaces made on 200 µm‐thick Si^[^ ^19^ ^]^ substrates are placed far apart (*d*  =  60 − 100 µm – green line), and then at a closer distance (*d*  =  30 − 50 µm – blue line). At resonance, an optical cavity is created due to reflections from the back and front ELC metasurfaces. At the larger cavity thickness, resonance remains at 900 GHz with a transmission depth at −51 dB and a Q‐factor of 56.25. However, as the cavity thickness is lowered, although the resonance frequency is unchanged, the transmission depth improves to −77 dB, yielding a massively improved Q‐factor of 642. Lower transmission depth at resonance signifies maximized reflection at resonance from the metasurface, leading to higher plasmonic interaction between the incoming THz wave and the free electrons of the resonating metallic metasurfaces. When the metasurfaces are very close (*d*  =  0 − 5 µ*m* – pink line), the average resonance frequency redshifts to 837 GHz, with a shallower transmission depth of −26 dB and a lower Q‐factor of 36.39. We hypothesize that there is an electromagnetic coupling between the two metasurfaces that dramatically enhances the Q‐factor at a particular distance, which may lead to significant resonant plasmonic enhancement and thus lead to higher sensitivity.

To further understand the electromagnetic coupling in the F2F metamaterial sensor, we simulated the electric fields at resonance emanating out from the metasurfaces at varying *d*. When the metasurfaces are kept at a large cavity distance (60–100 µm), the front metasurface shows a high intensity of resonant electric field pushing outward, indicated by the red‐colored field lines in Figure 1c. The back‐metasurface shows comparatively lower intensity of resonant electric fields (blue‐cyan streamlines). In this configuration, incoming THz radiation from the spectrometer first hits the resonant front‐metasurface through the front‐substrate, thus maximizing THz reflected radiation from the resonant metasurface into the substrate, generating highly intense electric field lines. Only a small portion of the transmitted THz radiation reaches the second metasurface, which then acts as its resonant field, showing with weaker electric field lines. At this distance, there is no evidence of direct coupling between the two electric fields emanating from the front‐and‐back‐metasurface. But there are reflections between front and back metasurfaces at resonance inside the cavity, due to which, the F2F MM blocks 6.24% more light compared to a single arrowhead metasurface (single MM) as calculated from simulated transmission parameters. Due to this multiple blocking of the THz radiation in the cavity at resonance, the resonance depth deepens (demonstrated in the green graph of Figure 1b). The 3D inset in Figure 1c demonstrates that there is no resonant field coupling from one metasurface to the other, which is also visible in the resonant electric field streamline plot.

When the metasurfaces are brought closer (d=30∼50μm), we observe very strong resonant interaction, as presented by the electric field lines (Figure 1d) that are directly electrically coupled between the two metasurfaces. In the 3D inset in Figure 1e, only the field lines that couple from one metasurface to the other are highlighted, along with the portion of the metasurface through which the field lines couple is shaded. The 3D inset shows that there is a high‐intensity electric field coupling between the inductor sidearms. The inductor sidearms of the 3D inset are shaded in deep orange with interconnecting red lines from one metasurface to the other, to indicate that the coupling is happening only through the outer sidearm of the metasurface. With closer analysis aided by the 3D‐modeled inset, a simpler representation of the resonating field streamlines, we can infer that the field lines emanating from the square sidearm structure (due to resonant current flow) of both metasurfaces are intercoupled, which indicates a strong electric field interaction between the two metasurfaces. We therefore see the most pronounced resonance dip due to intense electromagnetic coupling between the two metasurfaces, along with a significant improvement in resonance quality (demonstrated in the blue graph of Figure 1b). This cavity length (*d*  =  30 − 50 µm) is quite large to couple the capacitive fringing fields of one metasurface to the other.

When the metasurfaces are positioned closer (*d*  =  0 − 5 µm), we observe a reorganization of the electric field lines (in red) connecting the arrowhead capacitor lips of the metasurfaces, whereas a less intense coupling (blue‐cyan field lines) is observed between the square sidearm structures (Figure 1e). To simplify and understand the coupling mechanism in this configuration, as given in the resonant field streamline plotted in Figure 1e, one top‐view inset and one 3D‐modeled inset are presented. The top‐view inset demonstrates that the majority of the coupling effect is across the capacitive gap of the metasurfaces. Since both the metasurfaces are shown on top of each other in the top view inset, we cannot determine exactly how the resonant electric field is coupled from the capacitor lips of one metasurface to the other metasurface. The 3D‐modeled inset demonstrates the true nature of coupling; it shows that there is a low‐intensity electric field coupling between the inductor sidearms, now shaded in light orange with interconnecting light orange lines, to indicate a weaker coupling through the inductor sidearms. A much stronger coupling is happening between the capacitive fringing fields shown in red lines from the capacitor lip of one metasurface to the other metasurface. The capacitor lips are shaded in deep orange for easier understanding. If the resonant field streamline plot of Figure 1e is carefully observed then also we will be able to see that the field lines of the top and bottom of the metasurface that are connecting the inductor sidearms are of less intensity, compared to the field lines connecting the capacitor lips from one metasurface to the other (fringing field coupling). Due to a very short cavity distance between the two metasurfaces, the resonant dipoles of the capacitor plates are coupled, and therefore, the effective capacitance of the entire metasurface is increased. This phenomenon red shifts the resonance frequency and shows reduced resonance quality (demonstrated in the pink graph of Figure 1b). Hence, in this configuration, the two metasurfaces are supposed to act as one metasurface with a larger capacitor.

We further simulated the magnitude of the resonant electric field at the centre of the cap‐gap for front and back metasurfaces by varying the spacing between the metasurfaces *d*. For *d*  =  60 − 100 µm, the electric field at the front‐metasurface saturates at 3 MV m^−1^, while the back‐metasurface field decreases. At *d*  =  30 − 50 µm, the field peaks at 6.44 MV m^−1^ for both metasurfaces. At closer proximity, *d*  =  0 − 25 µm, the field intensity at both metasurfaces declines rapidly together until the metasurfaces are attached (*d*  =  0). We simulated several cavity distances, starting from *d*  =  0 to *d*  =  100 µm (detailed simulations given in Section S1, Supporting Information). At *d*  =  0, i.e., no cavity, the resonance frequency redshifts up to 700 GHz, which then gradually blueshifts to the pristine resonating frequency of a single MM, till *d*  =  10 µm. The electromagnetic coupling begins inside the cavity at *d*  =  30 µm, enhancing resonance depth and Q‐factor. This coupling effect remains the same up to *d*  =  50 µm. Beyond *d*  =  50 µm, the emanating electric fields start to decouple, reducing the resonant Q‐factor in the process. Although the enhanced blocking effect of incident THz radiation at resonance remains due to the creation of the optical cavity between two metasurfaces at *d* > 0 µ*m*, optimal coupling and resultant enhanced plasmonic interaction happen only at a specific *d*. Therefore, we demarcate three coupling zones (marked in cyan, green, and red shades at Figure 1f): first, no significant coupling inside the cavity when the metasurfaces are far apart (*d* > 50 µm), second, optimal coupling for closer distances (50 µm > *d* > 30 µm), and third, a unified metasurface for *d* < 30 µm. At *d*  =  40 µm, the transmission spectra comparison between a single MM and the electromagnetically coupled F2F MM (Figure 1g) reveals that the single MM has a resonance dip of −23 dB at 900 GHz (black line), while the F2F MM reaches a considerably lower resonance dip of −77 dB, at the same resonance frequency. Moreover, at *d*  =  40 µ*m*, the electric field peaks to a massive 6.4 MV m^−1^ at both the front and back metasurfaces, indicating strong electromagnetic coupling. Contrastingly, for *d*  =  100 µm, the front‐metasurface shows a resonant field of 2.8 MV m^−1^, while the back has only 0.1 MV m^−1^, signifying zero coupling between the metasurfaces (see Section S1, Supporting Information). This enhanced blocking of incident THz wave, indicating plasmonic enhancement at *d*  =  40 µm also has a direct correlation with enhanced dielectric sensitivity.^[^ ^19^, ^32^ ^]^ Although we have only presented numerical modeling and not analytical modeling for this phenomenon, we have drawn an interesting analogy of this coupling phenomenon to the wireless power/energy transfer model, details of which are given in Section S1 (Supporting Information).

### 2.2 Simulating the Dielectric Sensitivity of Optimally Coupled Metamaterials

**Figure 2:**
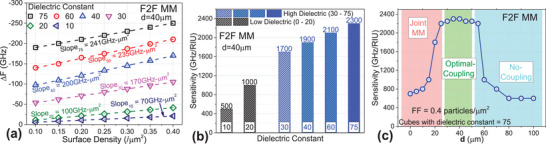
a) Simulated Δ*F* for varying dielectric constant and varying surface density (fill factor) is shown for F2F MM at the optimal coupling distance of 40 µm. The linear‐fitted slopes help to calculate sensitivity values. b) The simulated sensitivity values calculated from (a) are shown for varying dielectric constant for F2F MM at the optimal coupling distance of 40 µm. c) The simulated sensitivity values are plotted for varying *d*, for a fixed dielectric constant of 75 and FF of 0.4 particles µm^−2^.

To understand the effect of plasmonic enhancement for optimal coupling configuration on dielectric sensitivity, we simulated placing a dielectric nanoparticle in the active areas (cap‐gap) of both front and back‐metasurfaces with varied fill factor (FF) in low density as well as varied dielectric constant (but same density on both metasurfaces). For details of the simulations with dielectrics, see Section S2 (Supporting Information). The simulated Δ*F* is shown in **Figure** **2**a. We can observe that for low dielectric constants (10 and 20), the Δ*F* reaches −21 and −41.6 GHz, respectively, for FF up to 0.4 particles µm^−2^. For higher dielectric constants (30, 40, 60, and 75), the Δ*F* reaches −105, −170.2, −210, and −250 GHz, respectively. Comparing the optimal distance of F2F coupling configuration to a single arrowhead MM, for dielectric constant of 40 and FF of 0.4 particles µm^−2^, we got Δ*F* of −30 GHz,^[^ ^19^ ^]^ signifying a ≈6 times Δ*F* increases. We convert the FF variation to differential dielectric constant variation to simulate sensitivity in the more commonly accepted GHz/RIU^[^ ^21^, ^22^, ^24^ ^]^ as shown in Figure 2b. For the optimal coupling configuration, we achieved a high dielectric sensitivity up to 1000 GHz RIU^−1^ for low dielectric (0 to 20), and up to 2300 GHz RIU^−1^ for high dielectric constant (30–75). These dielectric constant values and the fill factors are deliberately chosen to match the type of organic and inorganic nanoparticles that we will use for impedance spectroscopy experiments.

When the dielectric sensitivity values are calculated for varying *d*, for a fixed dielectric constant of 75 and FF of 0.4 particles µm^−2^ (Figure 2c), we observe that the sensitivity remains constant at 500 GHz RIU^−1^ when there is no coupling between the two metasurfaces. Upon reaching the optimal coupling distance, the sensitivity increases rapidly and then remains constant within 2200 to 2300 GHz RIU^−1^. When the F2F MM at a very close proximity acts as a joint MM, the sensitivity starts dropping gradually, till 600 GHz RIU^−1^ when the metasurfaces are exactly touching each other. This simulation helps us to predict that the dielectric sensitivity will be stable in the optimal coupling distance range.

### 2.3 Experimental Validation of Optimal Electromagnetic Coupling Distance

**Figure 3:**
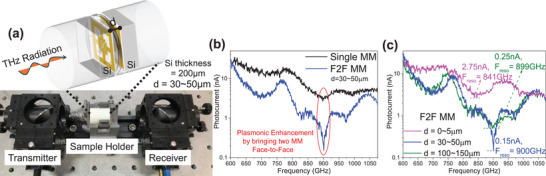
a) Experimental setup of THz impedance spectroscopy is shown along with the 3D printed hollow cylindrical capsule with the spacer. b) Experimental transmission photocurrent is plotted showing enhancement of plasmonic interaction, by a drastic increase of resonance depth, of our F2F MM, in optimal coupling distance of 30–50 µm. c) Experimental transmission photocurrent showing variation of F_reso_ and transmission depths at resonance with varying *d*.

Our experimental setup for transmission spectroscopy is shown in **Figure** **3**a. The THz spectroscopy setup consists of a Toptica Tera Scan 1550 linearly polarized spectrometer with a transmitter and receiver mounted with collimating parabolic mirrors (see Section S3, Supporting Information for details of the THz spectrometer). The metamaterial chip, consisting of a plurality of arrowhead structures arranged periodically is fabricated on 8‐inch Si wafers, then mechanically polished to achieve a 200 µm thickness and diced into 8 × 8 mm dies (see Section S4, Supporting Information for details of the thinning process, and Section S5, Supporting Information for details regarding fabrication procedures). To achieve the F2F configuration, a hollow cylindrical capsule fitting the dimensions of the metamaterial die is 3D printed with 40 µm spacer in the middle of the cylinder. This spacer is groove‐shaped (like a ring) that separates the two metasurfaces, as demonstrated in Figure 3a. Two metamaterial dies are inserted into the hollow cylinder from each end, maintaining a 30–50 µm distance between the two metasurfaces, which is then placed onto the sample holder for impedance spectroscopy.

We had demonstrated electromagnetic coupling between metamaterial resonance in the THz spectral range with substrate's FP oscillations, which degrades the metamaterial resonance quality factor as well as the dielectric response and sensitivity of the metamaterial, dramatically.^[^ ^19^ ^]^ A mechanism of decoupling the FP oscillations from the metamaterial resonance, by introducing longer FP oscillation periodicity, involved thinning down the substrate significantly via controlled mechanical polishing from 725 µm (typical thickness of 8‐inch wafer) to 200 µm. Such decoupling led to increased electric field density on the metamaterial surface, along with significantly enhanced plasmonic interaction on the metasurface. This led to a fivefold increase in the sensitivity of the dielectric response of the metamaterial, compared to conventional 725 µm‐thick substrates.^[^ ^19^ ^]^ We utilized this concept and did all our experiments on thinned 200 µm Si substrates only.

Figure 3b compares the transmission photocurrent for single and F2F metamaterials in an optimal coupling configuration. Both have the same resonance frequency; the single metamaterial shows a resonance depth of 3.22 nA, while the F2F configuration reaches 0.15 nA, indicating similar plasmonic enhancement as predicted in Figure 1g, which proves the hypothesis of electromagnetic coupling between the metasurface at this configuration. Upon comparing the transmission photocurrent with varying *d* (Figure 3c), for *d*  =  100 − 150 µm, we get a resonance frequency *F_reso_* at 899 GHz with depth reaching 0.25 nA, and Q‐factor of 57.29. For *d*  =  30 − 50 µm, we get *F_reso_* at 900 GHz with depth reaching 0.15 nA, and Q‐factor of 549. For *d*  =  0 − 5 µm, we get red‐shifted *F_reso_* at 841 GHz, with depth reaching 2.75 nA, and Q‐factor of 31.82. The resonance frequencies and Q‐factor values satisfy the simulation predictions (Figure 1b–e), signifying the three cavity thicknesses of electromagnetic coupling of the two metasurfaces. In all the cavity thicknesses, the resonance depth is significantly improved compared to single MM due to the resonant reflections between the two metasurfaces.

Therefore, as predicted in the simulations, the enhanced blocking of incident THz radiation by the resonant optical cavity is valid for all *d* > 0 µ*m*, with optimal coupling occurring only at a specific *d*. Moreover, since these periodic metasurfaces operate under resonant conditions, coupling occurs between the cumulative electromagnetic field of both metasurfaces. Therefore, there is no need for perfect alignment of the two metasurfaces to achieve this coupling (explained in detail in Section S6, Supporting Information). Figure 3d illustrates the transmission photocurrent at optimal coupling when the metasurfaces are carefully aligned, the back‐metasurface rotated 30°, and displaced 2 mm upward. Notably, only the F2F configuration is capable of optimal electromagnetic coupling; reversing one metasurface (back‐to‐face) eliminates any probability of coupling (see Section S7, Supporting Information). We plot Figures 3c and 1b on the same graph to demonstrate the similarity of resonance frequencies in all the coupling configurations between simulations and experimental results in Section S8 (Supporting Information).

The optimal coupling cavity length (30–50 µm) is not related to the incident THz wavelength, but the shape and the size of the metasurface, and its resonance strength. The near‐field coupling/interaction is happening at resonating frequencies, which are, by themselves, dictated by the geometry of the metamaterial.^[^ ^22^ ^]^ Due to the plasmonic interaction between the THz wave and the resonating metamaterial, strong electromagnetic field lines are formed due to capacitive dipole formation, which emanate from the metasurface. These resonant fields (as plotted in Figure 1c–e) dictate the nature of coupling between two metasurfaces. If the metasurface is made up of more inductive and capacitive elements, or an asymmetry is added for dark‐mode ignition^[^ ^37^, ^38^, ^39^, ^40^ ^]^ (more complex structure), the strength of these emanating field lines will vary, and so the optimal coupling cavity length will also vary. The evanescent decay length will vary with the resonance strength of the metamaterial, and the optimal coupling cavity length will always be shorter than the evanescent decay length to ensure proper electromagnetic coupling. Cavity resonance conditions will not affect, since the cavity length of 30–50 µm for optimal coupling configuration is much less than the incident THz wavelength (which is ≈300 µm in air).

### 2.4 Experimental THz Impedance Spectroscopy of Nanoparticles and Dielectric Sensitivity

For impedance spectroscopy measurements, we used Bovine Serum Albumin (BSA) form very low (10^−6^%) to moderate (10^−3^%) concentrations, glucose (sugar solution) with varying concentration from 20 to 150 mg DL^−1^, inorganic nanoparticles line ZnO (50 nm sized) and PbN and salt (0.9% saline solution), to check the dielectric response among different organic and inorganic substances. The protein, sugar, and salt are deposited on both the metasurface by drop and dry methods, and the nanoparticles are sprinkled homogeneously. See Section S9 (Supporting Information) for more details regarding the analyte preparations. In addition, an accurate mapping of the spatial distribution of the dielectric substance is not required and has no real effect on the dielectric performance of our metamaterial sensor, provided the dielectric particles fall on the active area (cap‐gap) for sensing, as discussed in Section S9 (Supporting Information).

**Figure 4:**
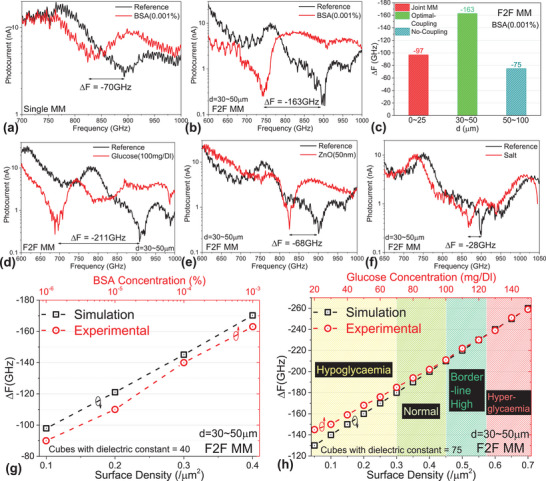
Experimental impedance spectroscopy measurements showing Δ*F* for the BSA dielectric for a) single MM, and b) F2F MM in the optimal coupling configuration. c) Bar chart showing experimental Δ*F* for varying *d*.Experimental impedance spectroscopy measurements showing Δ*F* for d) 100 mg DL^−1^ of glucose, e) 50nm‐sized ZnO nano powder and f) 0.9% saline solution, for F2F MM in optimal coupling configuration. g) Simulated Δ*F* (black data points) with varying surface density (FF 0.1 to 0.4 µm^−2^) of cubes with dielectric constant 40, is plotted on the same graph with experimental Δ*F* (blue data points) with BSA concentration densities 10^−3^ to 10^−6^% for F2F MM at optimal coupling configuration. h) Simulated Δ*F* (black data points) with varying surface density (FF 0.05 to 0.7 µm^−2^) of cubes with dielectric constant 75, is plotted on the same graph with experimental Δ*F* (blue data points) with glucose concentration densities 20 to 150 mg DL^−1^ for F2F MM at optimal coupling configuration.

As a baseline, Δ*F* for single MM on 200 µm‐thick Si substrate and 10^−3^% BSA concentration as a dielectric material is only −70 GHz (**Figure** **4**a). Contrastingly, for the optimal coupling configuration for F2F MM, the Δ*F* increases to −163 GHz for the same 10^−3^% BSA concentration (Figure 4b). For reference, the Δ*F* for the optimal coupling configuration for F2F MM fabricated on a standard 725 µm substrate (8‐inch wafer) is −33.29 GHz (see Section S10, Supporting Information). Additionally, we observe the improvement in resonance quality due to the optimal electromagnetic coupling from Figure 4a,b, which is independent of the spectral shift. For the other cavity thicknesses, Δ*F* for *d*  =  0 − 25 µm is averaged at −97 GHz, whereas, for *d* > 50 µm its ‐75 GHz analogous to single MM (Figure 4c). This impedance spectroscopy study experimentally validates the three coupling zones with the help of the relation of Δ*F* with *d*. Interestingly, If the BSA is deposited only on the front‐metasurface or the back‐metasurface we get Δ*F*  =   − 73 GHz exactly analogous to the single MM 200 µm thick Si substrate (see Section S11, Supporting Information). For sugar as a dielectric, the optimally coupled F2F MM gives Δ*F* of −211 GHz for 100 mg DL^−1^ of glucose solution (Figure 4d). In case of nanoparticles, the F2F MM gives Δ*F* of −68 GHz for 50 nm sized ZnO (Figure 4e), and −61 GHz for PbN. For salt 0.9% saline solution (particle of low dielectric constant), we get Δ*F* of −28 GHz (Figure 4f), signifying, higher dielectric constant achieves larger Δ*F*. BSA has a dielectric constant of 40,^[^ ^41^ ^]^ sugar solution has a dielectric constant of 70–80,^[^ ^42^, ^43^ ^]^ and most inorganic nanoparticles including salt having dielectric constant <20.

Figure 4g compares the simulation results with the statistically averaged experimental Δ*F* results for varying BSA concentrations from 10^−3^% diluting up to 10^−6^%, and for varying FF's (from Figure 2a) with different X‐axes on the same graph. From the data in Figure 4g we can calibrate the experimental Δ*F* values in a way such that Δ*F* for 10^−3^% BSA, 10^−4^% BSA, 10^−5^% BSA, and 10^−6^% BSA correlates with the simulated Δ*F* values for FF of 0.4, 0.3, 0.2, and 0.1 µm^−2^ respectively, for particles (emulated as cubes) with dielectric constant of 40. This correlation has been previously established in our published works.^[^ ^19^, ^20^, ^21^, ^32^ ^]^ We can obtain a similar linear trend of Δ*F* with identical slope implying proper correlation between simulations and experiments. Similarly, in Figure 4h we compare statistically averaged experimental Δ*F* for varying glucose concentrations from 20 mg DL^−1^ to 150 mg/Dl with simulated Δ*F* of varying FF from for 0.1/µm^2^ to 0.7/µm^2^ with particles of dielectric constant of 75. Upon correlating the glucose concentration to human blood sugar levels, we segregated the Δ*F* among four zones.^[^ ^42^, ^43^, ^44^, ^45^ ^]^ For the hypoglycaemia zone (Δ*F*  =   − 145 to − 185 GHz) there is a small, discrepancy of <10 GHz between the simulation and experimental results. In the normal (Δ*F*  =   − 185 to − 211 GHz), borderline high (Δ*F*  =   − 211 to − 235 GHz), and hyperglycaemia zone (Δ*F*  =   − 235 to − 259 GHz), we have good correlation. Using these correlations, we compare the linear slopes of experimental and simulated Δ*F* with respect to dielectric concentration and FF, respectively, which is then used to calculate sensitivity. If the linear slope of both simulations and experiments correlates, we can comprehend that the simulated sensitivity values in Figure 2b reasonably correspond to the experimental sensitivity values. The equivalent FF value in simulations is then converted to differential change in dielectric constant^[^ ^21^, ^22^, ^24^ ^]^ to obtain sensitivity in GHz/RIU (this method has been used in our previous works,^[^ ^21^, ^22^, ^24^, ^46^, ^47^ ^]^ and also discussed in detail in Section S12, Supporting Information).

**Figure 5:**
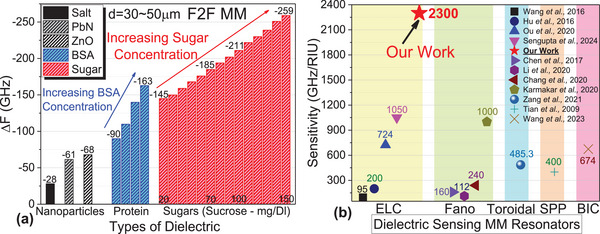
a) Bar chart showing different dielectric responses for nanoparticles, proteins, and sugars, for our F2F MM at optimal coupling configuration. b) The dielectric sensitivity of our F2F MM in optimal coupling configuration is compared with different types of dielectric sensing MM resonators. All the referenced works have a sensitivity magnitude label, where our work, denoted with a red coloured star, has a sensitivity of 2300 GHz RIU^−1^.

**Figure** **5**a summarizes the impedance spectroscopic measurements in bar charts (each data point statistically averaged), showing the range of Δ*F* for various substances. Inorganic nanoparticles exhibit shifts up to −70 GHz, while proteins’ (BSA) shifts range from −90 GHz (very low concentration) to −163 GHz (moderate concentration), and sugars range from −145 GHz for 20 gm DL^−1^ to −259 GHz for 150 gm DL^−1^. This establishes clear Δ*F* boundaries between low permittivity inorganic nanoparticles and high permittivity organic substances and sugars. Most practical applications study organic substances and sugars with dielectric constants ranging from 30 to 80. In Figure 2b, we calculated the sensitivity values ranging from 1700 to 2300 GHz RIU^−1^ for this dielectric range. Figure 5b compares this sensitivity with various types of metamaterial resonators, such as ELC,^[^ ^21^, ^44^, ^48^, ^49^ ^]^ Fano,^[^ ^50^, ^51^, ^52^, ^53^ ^]^ toroidal,^[^ ^25^ ^]^ surface plasmon polariton (SPP),^[^ ^25^ ^]^ and quasi‐bound states in continuum (quasi‐BIC)^[^ ^54^ ^]^ sensors, which mainly detected organic substances, cells or sugar solution. Compared to the best sensitivity recorded to date (1050 GHz RIU^−1^), which is also of an ELC resonator, in the F2F architecture, we increase sensitivity by 2.2‐fold, reaching 2300 GHz RIU. Interestingly, the sensitivity values we calculated for low dielectrics (<20) in Figure 2b are now comparable to most of the sensitivity recorded in literature for high dielectrics (>30).

On another interesting note, when we analyse the dielectric response of the metamaterial on the standard 725 µm thick Si substrate (for 8‐inch wafer), it becomes evident that this observed sensitivity and plasmonic enhancement is a result of combined effect of thinning of the substrate^[^ ^19^ ^]^ and achieving optimal electromagnetic coupling of the two metasurfaces. For the 725 µm thick substrate 10^−3^% BSA gives Δ*F* of only −39 GHz.^[^ ^19^ ^]^ However, when the same sample is thinned to 200 µm increases the Δ*F* to −70 GHz (Figure 3a). In case of the F2F optimal coupling configuration, standard 725 µm thick Si yields a Δ*F* of −102 GHz, but thinning the substrates to 200 µm increases the Δ*F* to −163 GHz as shown in Figure 3b. Upon comparing the sensitivity values, we observe a 23‐fold enhancement in sensitivity compared to the single‐MM arrowhead ELC resonator fabricated on 725 µm‐thick substrate, which has a sensitivity of only 100 GHz RIU^−1^.^[^ ^20^, ^21^ ^]^ A table to summarize all the sensitivity parameters with respect to types of dielectrics is given in Section S13 (Supporting Information). A discussion comparing the complexity, cost, and scalability of our F2F MM sensor with some other metamaterial architectures is added in Section S14 (Supporting Information).

## 3 Conclusion

This work demonstrates a unique electromagnetic coupling mechanism between two (F2F) passive ELC resonating metamaterials in a resonating THz optical cavity, which results in sensitivity enhancement. The coupling is triggered when the two metasurfaces are brought in optimal proximity to one another. Based on the distance between two metasurfaces, we demarcated three cavity thicknesses in terms of coupling, with best sensitivity achievable from the optimal coupling zone, in particular, a distance of 30–50 µm. There is an intense coupling of resonant electric fields in this zone, as two metasurfaces constructively interfere, inducing high plasmonic enhancement. Besides the high‐Q factor resonance of 549, this optimal coupling region is copiously stable for any misalignment, and dielectric sensitivity remains unaltered for small deviations in *d* between the two metasurfaces. This sensor detects a variety of inorganic and organic nanoparticles as well as sugar in ultra‐low concentrations. In comparison to a single‐MM arrowhead ELC resonator fabricated on a standard 725 µm‐thick substrate, we achieve 23 times higher dielectric sensitivity. In fact, compared to latest works on THz dielectric sensors (2016–2024), our work demonstrates one of the highest sensitivities recorded for any ELC resonator reaching up to 2300 GHz RIU^−1^, which means that a 1 GHz of spectral shift can be brought by only a change of 0.189 µ dielectric constant (permittivity). Most of the previous works published to date can only reach up to 1 millichange of dielectric constant per 1 GHz of spectral shift. Although the effect of the optimal electromagnetic coupling plays a greater role in dielectric sensitivity enhancement, the contribution of thinning the substrate cannot be ruled out. This highly sensitive, fully CMOS‐compatible sensor is ideal for ultra‐sensitive dielectric sensing applications, including nanoparticle and biomolecule characterization and the detection of viruses and bacteria, or any other biological samples.

## Conflict of Interest

The authors declare no conflict of interest.

## Supporting information



Supporting Information

## Data Availability

The data that support the findings of this study are available in the supplementary material of this article.
