# Supplementary material for: Electromagnetically Coupled Resonant Face‐to‐Face Double‐Layer Metamaterial for Highly Sensitive THz Impedance Spectroscopy
Source: Adv Sci (Weinh). 2025 Aug 26;12(38):e04331. doi: 10.1002/advs.202504331 (PMC12520489; doi:10.1002/advs.202504331)
Supplement: Supplementary file 1 — Supporting Information [file ADVS-12-e04331-s001.pdf]

ADVANCED SCIENCE
Open Access

Supporting Information

for *Adv. Sci.*, DOI 10.1002/advs.202504331

Electromagnetically Coupled Resonant Face-to-Face Double-Layer Metamaterial for Highly Sensitive THz Impedance Spectroscopy

*Rudrarup Sengupta, Heena Khand and Gabby Sarusi**

Electromagnetically Coupled Resonant Face-to-Face Double-Layer Metamaterial for Highly Sensitive THz Impedance Spectroscopy

Supporting Information

Rudrarup Sengupta[§], Heena Khand[§], and Gabby Sarusi*

Department of Photonics and Electro-Optics Engineering, School of Electrical and Computer Engineering,

Ben-Gurion University of the Negev, Beer Sheva, Israel.

*Correspondence author, Orcid ID: 0000-0001-6717-2235; email: sarusiga@bgu.ac.il

[§]Equally contributed to this work.

1: Resonance characteristics at different cavity distances

Here we simulate several cavity distances parametrically, starting from $d = 0$ to $d = 100\ \mu m$ with very short steps. At $d = 0$ i.e., no cavity, the resonance frequency starts from 700 GHz (when the capacitance and inductance of the metasurface is increased), which then gradually blueshifts to the pristine resonating frequency of a single MM, till $d = 10\ \mu m$, shown in figure S1(a). From $d = 0$ to $d = 10\ \mu m$ we observe gradual blue-shift of the metamaterial resonance due to weakening of the capacitive fringing field coupling. The fringing fields of the resonant capacitors get completely un-coupled at $d = 30\ \mu m$, beginning a pure plasmonic interaction due to electromagnetic coupling between the inductor sidearms, enhancing resonance depth and Q-factor. This coupling effect remains the same up to $d = 50\ \mu m$, as shown in figure S1(b). Beyond $d = 50\ \mu m$, the emanating electric fields start to decouple, shown by reducing quality factor at resonance, in figure S1(c). Although the enhanced blocking effect of incident THz radiation at resonance remains due to creation of the optical cavity between two metasurfaces at any $d > 0\ \mu m$, optimal coupling and resultant enhanced plasmonic interaction happens only at a specific range of d $(30\sim 50\ \mu m)$. All the simulated graphs for various distances are clearly marked in figure S1, describing this phenomenon.

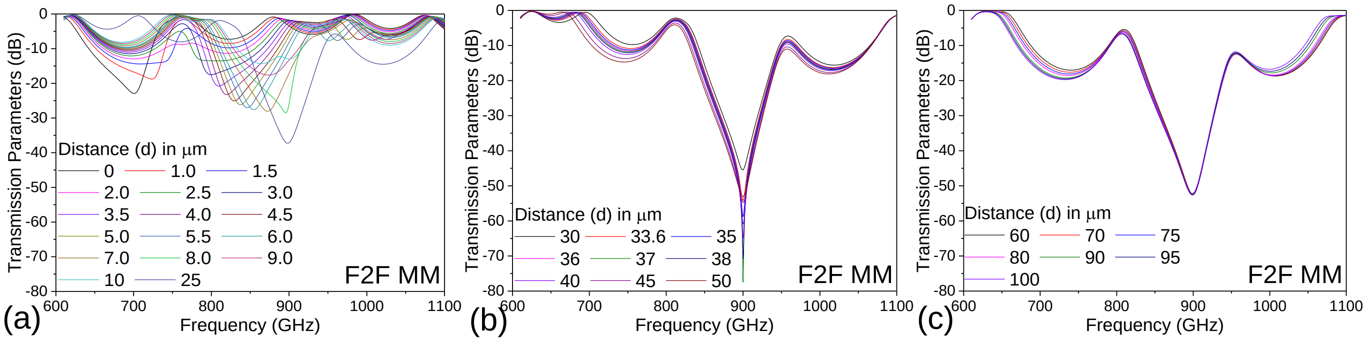

Figure S1. Simulated transmission parameters are plotted for varying d, (a) $d = 0\ to\ 25\ \mu m$, (b) $d = 30\ to\ 50\ \mu m$, and (c) $d = 60\ to\ 100\ \mu m$.

Now we plot the simulated electric field plotted along the length of the substrate starting from the centre of the metasurface's active area, for $d_1 = 40\ \mu m$ in blue line and $d_2 = 100\ \mu m$ in green line

showing the electric field magnitudes at both metasurfaces. In a distance range of 30-50 μm, optimal coupling with the best field enhancement occurs at $d = 40\ \mu m$, where the electric field peaks to 6.4 MV/m at both metasurfaces, indicating strongest electromagnetic coupling. For $d = 100\ \mu m$, the front-metasurface shows a resonant field of 2.8 MV/m, while the back has only 0.1 MV/m, signifying zero coupling between the metasurfaces.

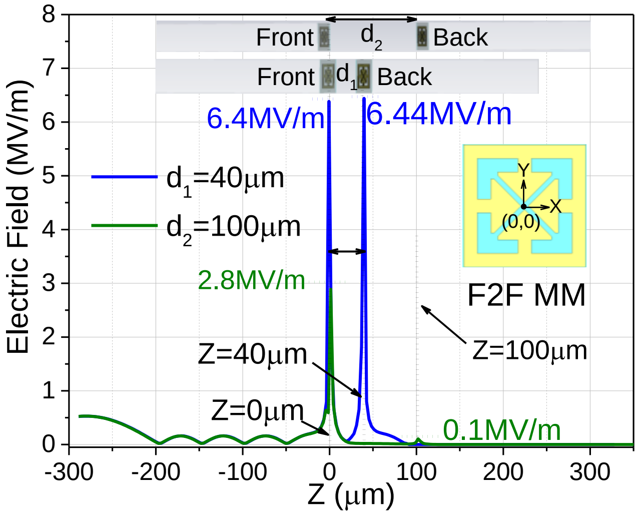

Figure S2. Simulated electric field plotted along the length of the substrate starting from the centre of the metasurface's active area, for $d_1 = 40\ \mu m$ in blue line and $d_2 = 100\ \mu m$ in green line showing the electric field magnitudes at both metasurfaces.

We are basically looking at two phenomena here, first, the strong plasmonic coupling at $d = 30{\sim}50\ \mu m$, and then a weaker plasmonic and stronger capacitive fringing field coupling for extremely close proximity of $d = 0{\sim}5\ \mu m$. Although we have not proposed any conventional mathematical model describing this phenomenon, we would like to draw an interesting analogy of this coupling phenomenon to the wireless power/energy transfer model. In case of an inductive wireless power/energy transfer, we generally observe that the coupling coefficient increases with an increase in the proximity between two indictor coils, which results in enhanced field coupling. A similar phenomenon is happening here under resonant THz illumination (which is acting like a power source). At $= 30{\sim}50\ \mu m$, the resonant inductor sidearms are electromagnetically coupled with each other strongly enabling plasmonic interaction between the two metasurfaces. The resonating LC's current flow extends from one metasurface to the other electromagnetically to achieve a perfect coupling. Now when the metasurfaces are brought at extremely close proximity of $d = 0{\sim}5\ \mu m$, stronger coupling happens between the fringing fields of the resonant capacitor lips, and the coupling between the inductor sidearm weakens comparatively. This phenomenon is analogous to a combination of inductive and capacitive power/energy transfer. But because the L and C components are parts of the same circuit and the electromagnetic coupling (analogous to energy transfer) is occurring at resonance, the two metasurface now begins to act as one enlarged metasurface with increased capacitance and inductance. This results in the effective capacitance and inductance of the entire

metasurfaces to be increased, thereby red-shifting the resonance frequencies. This is an intuitive comparison of our work to a well-known theoretical model.

2: Details regarding the simulation with dielectrics

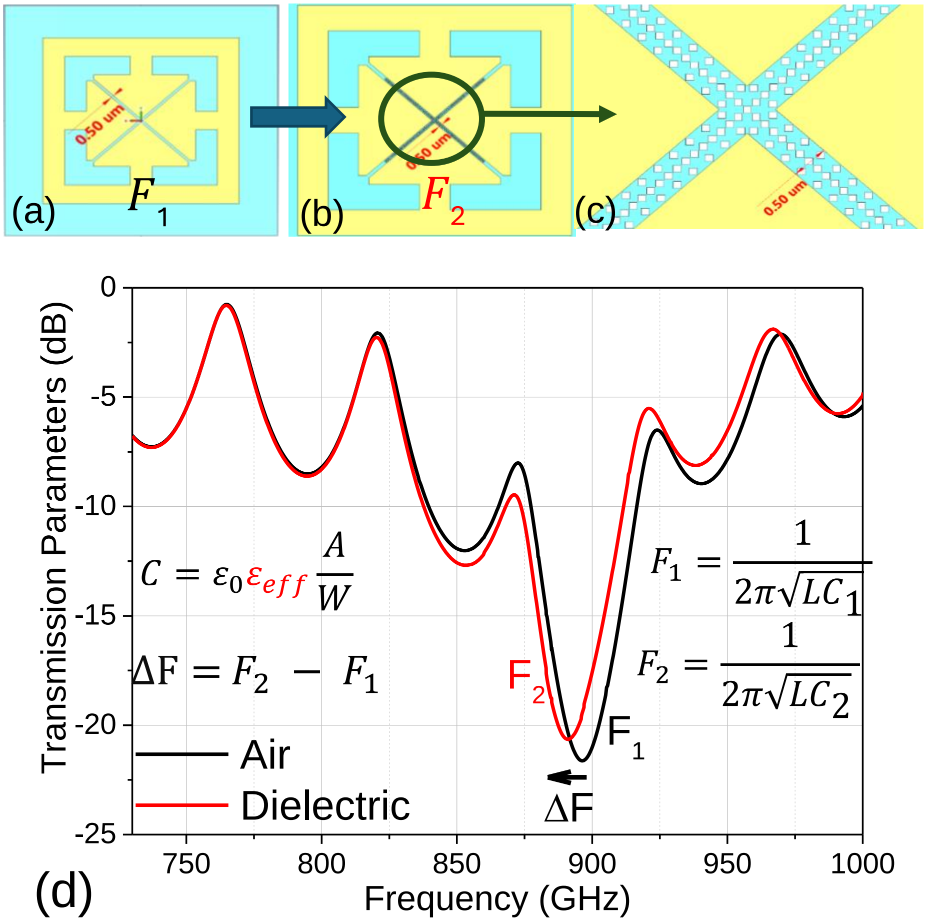

Figure S3. (a) shows the structure of 4 arrowhead MM with 0.5µm cap-gap without any dielectric particle. (b) Illustrates the MM structure with cubic dielectric particles sized 100nm in length and (c) shows a zoomed picture. (d) The transmission spectra of MM resonance for (a) and (b) cases show red-shift in frequency for dielectrics placed in cap-gap by the formulae shown.

The 3D simulation was done extensively using CST studio suite for analysing the Metamaterial (MM) along with its full system of chip housing. First, we build and analyse the 4-arrowhead MM structure of the chip (figure S3(a)) which consists of a plurality of antenna structures.

To know the dielectric response of the MM, we design nanoparticle cubes (as analytes) of length 100 nm and specific dielectric constant to distribute them within the capacitor-gap (cap-gap) of the MM with a specific fill factor (FF) as shown in figure S3(b-c). Resonating frequency of the MM structure (in vacuum) is shown in the S-parameters in the transmittance graph shown in figure S3(d). The distribution/concentration of the cubes (figure S3(c)) and their dielectric constant can be varied accordingly. The presence of analytes in the cap-gap (in comparison to the structure without analyte in figure S3(a)) will result in a red shift of the resonance frequency, due to an increase in the capacitance of the capacitors; the

shift in frequency ΔF (dielectric response) is shown by figure S3(d) and the equations are added to understand the red-shift in frequency. Note that the simulation method was such that dielectrics were deliberately put in low concentration/quantity and nanoparticle sized. Also, note that, in figure S3, cap-gap of 0.5 µm is illustrated, which has a resonance frequency of ~900 GHz.

In simulation we vary the fill factor of the cubes placed in the active area to get spectral shift for different concentrations. In our work, we correlate between simulated and experimental ΔF data with respect to varying dielectric concentration. This method offers an opportunity to calculate and predict dielectric response by simply performing simulations with different fill-factor values. In this process, we initially calibrated the experimental dielectric concentrations against the equivalent fill-factor value in the simulations for a series of data points so that we can achieve identical ΔF values in simulations and experiments. After this step, we extrapolate and predict the ΔF for extremely low concentration dielectrics. That is why in figure 4g and h we compare the simulation and experimental ΔF in an X-X-Y graph, so that the ΔF values can be qualitatively compared with respect to varying fill factor (simulations) and dielectric concentration (experiments).

3: Information about THz spectrometer used

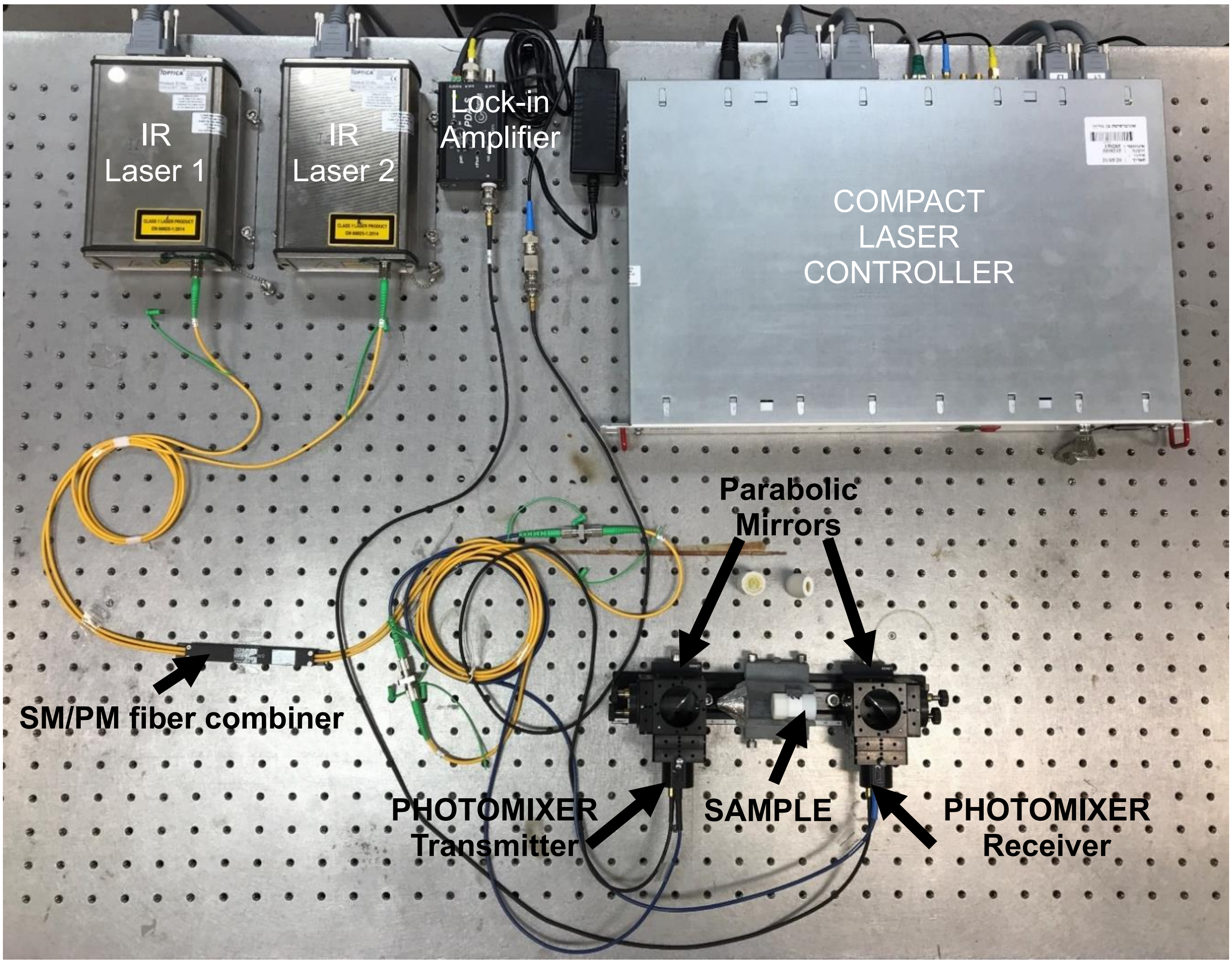

Figure S4. Snapshot of our entire THz scanning system used to screen coronavirus and related particles.

We used a linearly polarized Toptica Systems TeraScan 1550 to record the transmittance spectra. This spectrometer has an InGaAs photomixer with a metal-insulator-metal heterostructure architecture. The photomixers use distributed laser feedback (DFB) technology to unite two temperature-controlled 1.5 um lasers with a minute difference in wavelength and obtain the envelope of the interference spectrum, termed the 'laser beat', which is in the THz domain[1]. This spectrometer works with a coherent detection scheme, where the second photomixer acts as the THz receiver. The incoming THz wave generates a voltage in the antenna, while the 'laser beat' modulates the conductivity of the photomixer[1]. The resulting photocurrent (which is the unit of the output spectra) is proportional to the amplitude of the THz electric field[1]. The entire setup is controlled by a microcontroller unit (MCU) based on a FPGA with an internal clock rate of 130 MHz to facilitate different programming operations[1]. Figure S4 shows our entire THz scanning setup for screening coronavirus carriers.

4: Polishing and thinning of substrate

We use the automatic and the manual polisher to thin down the conventional 725 µm thick Si substrate to 200 µm thickness. Note that we covered the antennas with a protective film to avoid any damage and polished the die from the backside. We put the chip on the stub as shown in figure S5 (b) such that the substrate is on the top side as shown in the picture. We use three different types of polishing paper grades to thin down the Si substrate and then to mirror-polish the surface. While working with the automatic polisher, we keep the minimum pressure for a longer time to thin accurately, and consequently mirror-polish in the manual polisher.

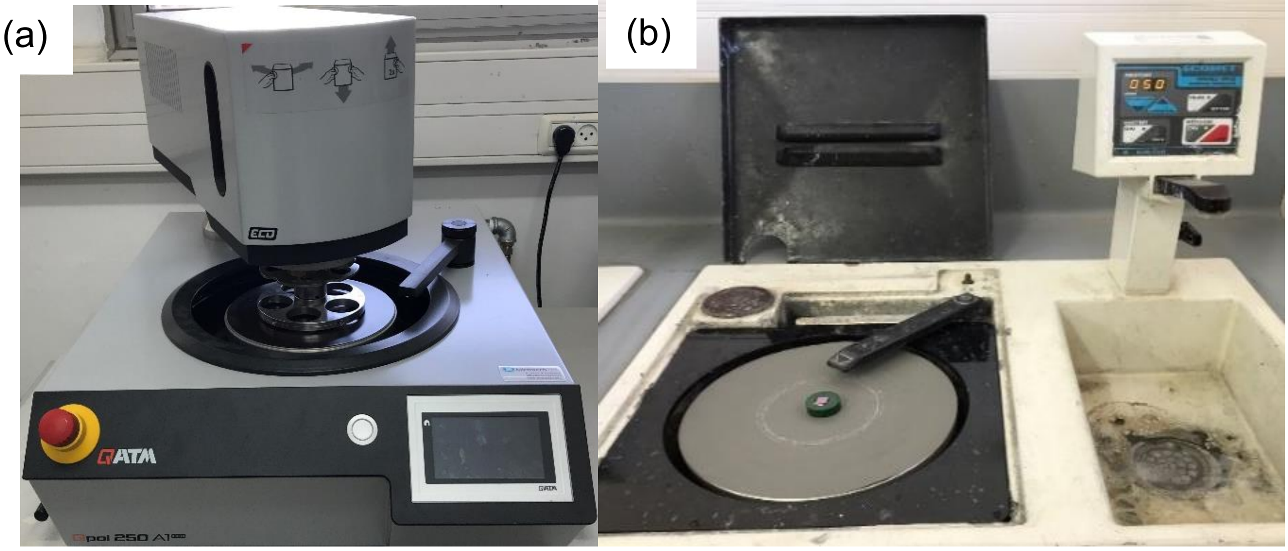

Figure S5. (a) Automatic polisher for thinning 725 µm thick Si to 200 µm (b) manual polisher used for thinning and polishing the chip placed (MM structures facing down) on the green stub as shown.

The automated mechanical back-polishing process of our metamaterial sensors with an error of ±25 µm. In this range, the metamaterial sensitivity remains the same. In our previously published paper

(reference number 19 of the main manuscript) we have demonstrated how the ΔF varies with respect to substrate thickness. We found out that the ΔF increases linearly from 500 µm Si thickness to 300 µm Si thickness and then starts to saturate after that. So, when we make slight errors in polishing the sample and the thickness varies as 200±25 µm, the resonance frequency and ΔF remains completely stable.

5: Fabrication methods

Cross-arrowhead MM structure with 0.5 µm cap-gap width used for the impedance spectroscopy measurements is fabricated on an 8-inch Si wafer having a standard thickness of 725±25 µm and diced into 8 mm squares chips, with each chip containing around 14,400 nano-antenna structures forming the MM surface. The chips are prepared by e-beam evaporation and standard lithography. Figure S6 shows a microscopic image of our fabricated four arrowhead LC resonant MM patterns. A 200 nm Aluminium film was deposited by e-beam evaporation to define arrays of electrical arrowhead resonators with a line width of 4 µm, outer dimensions of 36 µm × 36 µm, and a cap-gap of 0.5 µm. The pitch between the elements is 50 µm.

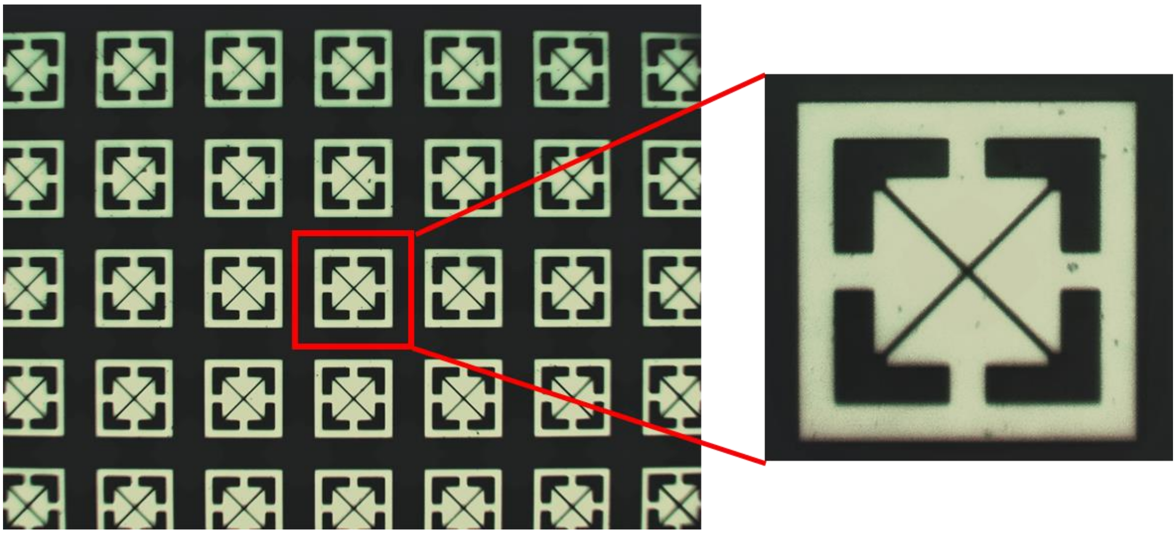

Figure S6. Microscopic image of the fabricated arrowhead LC resonant MM structure on Si chip.

Fully developed and tested nanolithography process can also have minor errors in the metasurface dimensions fabricated. But this will not impact on our dielectric sensing performance. For example, if the cap-gap has and error of ±0.1 µm, or the metasurface outer dimensions or the capacitor lips have a similar error, the resonating frequency of the metamaterial senor will vary by a small margin. But we have ensured to take both the metamaterial chips of the F2F MM sensor from the same processed wafer, so that there is no anomaly between the resonating frequencies of the chips placed in F2F configuration.

6: Stable alignment of F2F MM simulation

In this simulation, we show variation in transmission photocurrent with varying alignment of front and back metasurfaces are shown of the F2F MM at optimal coupling configuration in figure S7, when the

metasurfaces are carefully aligned (black line), the back-metasurface rotated 30º (pink line), and displaced 2 mm upwards (blue line). We achieve identical resonant characteristics with same F_{reso} and Q-factor.

Since our metamaterial is super symmetric, the optimal coupling condition is completely stable under rotational misalignment. For displacement misalignment, we did a displacement of 2 mm only, and our metamaterial die is of the size 6 mm by 6 mm. Therefore, we misaligned the chip by 30% of its size, which should be in-range for any accidental misalignment. But we have observed that if we misalign the die by 4 mm or more (more than 60% of misalignment), the quality factor of the resonance degrades, indicating the coupling between two metasurfaces have weakened. Misalignment above 4 mm, results in the two metasurface acing as separate uncoupled entities with behavior analogous when the when the metasurfaces are kept at a large distance (60~100 µm). Our spectrometer has a THz beamwidth of more than 10 mm, so the receiver can see the effect of both the resonating metasurfaces even though they are highly misaligned.

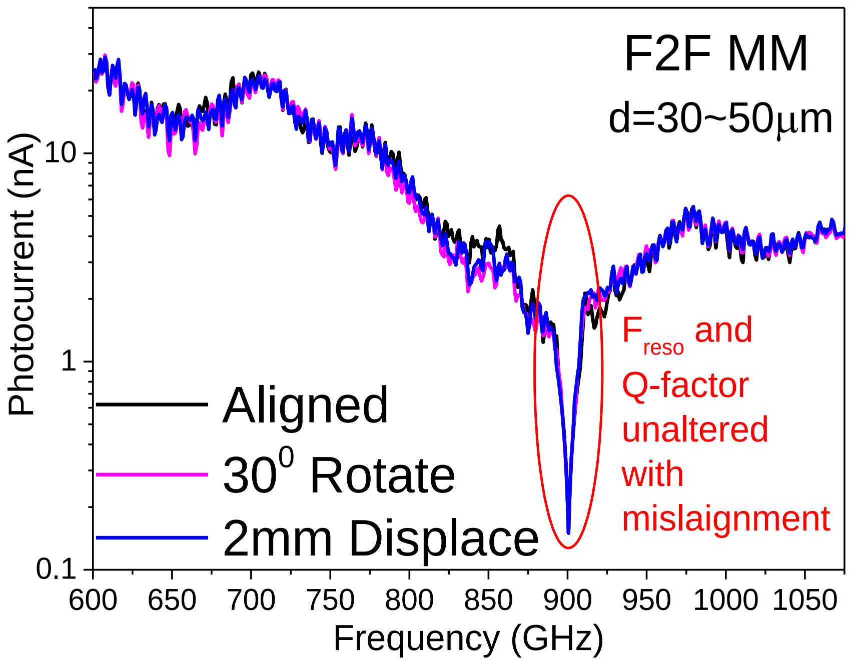

Figure S7. Transmission photocurrent plotted with varying alignment of front and back metasurfaces.

7: Back-to-Face (B2F) coupling MM simulation

Here we experimentally demonstrate that, only the F2F configuration is capable of optimal electromagnetic coupling. Experimental transmission photocurrent is plotted in figure S8 for single MM (black line), F2F MM (blue line) and back-to-front (B2F) configuration MM (pink line) for $d = 30 \sim 50\ \mu m$. Reversing one metasurface (B2F) strikingly reduces the resonance Q-factor, which is a proof of elimination of any probability of coupling.

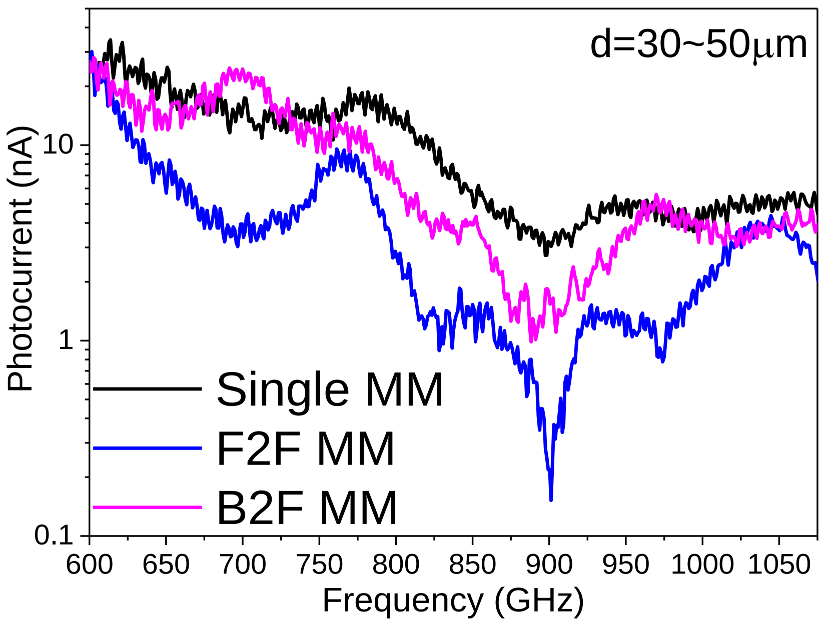

Figure S8. Experimental transmission photocurrent plotted for single MM (black line), F2F MM (blue line) and back-to-front (B2F) configuration MM (pink line) for $d = 30 \sim 50\ \mu m$.

8: Simulation and experimental spectra comparison

In figure S9, the left Y-axis denotes the experimental photocurrents (in logarithmic scale) and the right Y-axis denotes the simulated S-parameters (in logarithmic scale dB). The simulated spectra are denoted in dashed lines whereas the experimental spectra are denoted by solid lines. The resonance regions for different cavity lengths are circled with the respective legend colors. We can observe here that the resonance frequencies for F2F MM with different cavity lengths are exactly same when simulations are compared with experiments. Also, the resonance frequency depths and the resonance shape (determining the Q-factor) are also more or less the same when simulations are compared with experiments, except for $d = 0{\sim}5\ \mu m$. Still, we achieve similar Q-factor of all simulations compared with experiments. This visual difference in the spectra is noticeable because of the nature of operation of our spectrometer.

Our spectrometer operates in frequency domain and gives its output in photocurrents. We receive higher levels of photocurrents for lower frequencies and lower levels of photocurrent at higher frequencies due to the specific characteristics of the receiving photodiode. That is why we see a slanted spectrum when we scan over a large range of frequencies. On the other hand, the simulated S-parameters are generally obtained in linear scale or on logarithmic scale (dB). A direct spectral comparison between the simulated and the experimental will not be feasible. That is why we have compared the resonance frequency and the Q-factor in between simulation (figure 1b) and experiment (figure 3c), instead of comparing every aspect of the spectra.

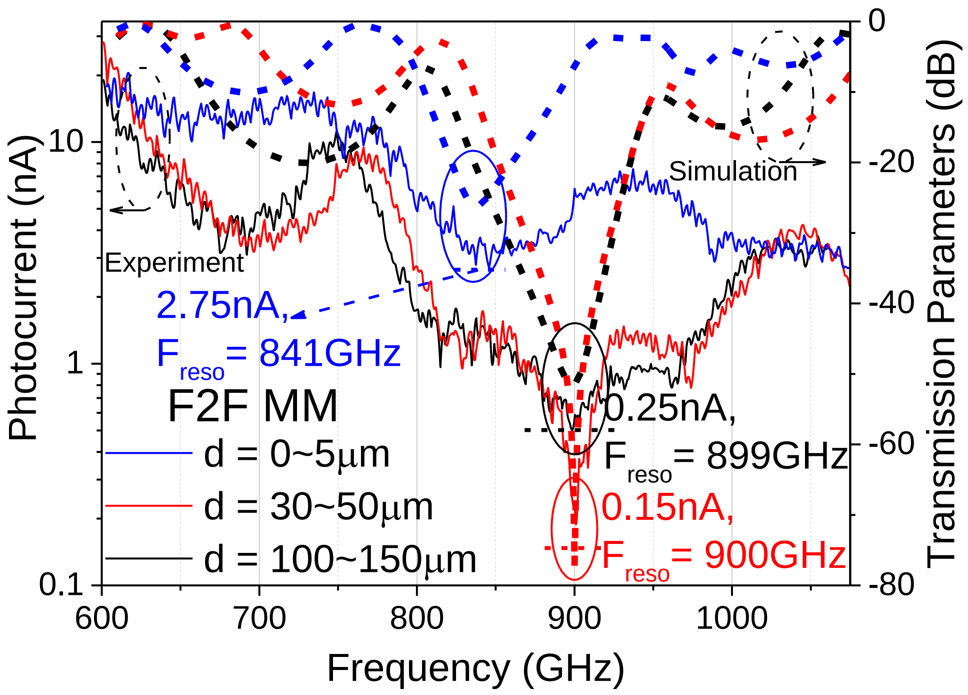

Figure S9. Comparison between experimental transmission photocurrent showing variation of F_{reso} and transmission depths at resonance with varying d and simulated transmission parameters showing variation of resonance frequency (F_{reso}) and transmission depths at resonance with varying d.

9: Preparation of analytes for impedance spectroscopy

For the THz impedance spectroscopy experiments, we used nanoparticles such as ZnO of 50 nm size and PbN as the nanoparticle analytes spread homogeneously on the MM chip surface. For higher dielectric and a more homogeneous distribution of analyte, we used protein nanoparticles of Bovine Serum Albumin (BSA), which is dissolved and diluted in saline solution or de-ionized (DI), water in an organized way for our experiments. To dilute the 140 gm of BSA particles, we use 1 Liter of 0.9% saline solution. This solution is then diluted to 0.001%, 0.0001%, 0.00001% and 0.000001%. (0.9% saline means that in 100 ml of DI water, we solubilize 0.9 mg NaCl). We put varying solutions of BSA with the help of a pipette measuring 20 μL on the MM chip surfaces and wait for the water to dry (drop and dry method). After this solution is perfectly dried on the surface, we check for the transmission spectra and subsequent dielectric response of the MM. Similarly, glucose solutions are made by dissolving sucrose in DI water in concentrations starting from 20 mg/Dl up to 150 mg/Dl. 20 μL of the solution is then deposited on the MM chip surfaces by drop and dry method. For measuring dielectric response of salt, we solubilize 0.9 mg NaCl in 100ml of DI water and use drop and dry method for impedance spectroscopy.

The spatial distribution of the sensing substance (dielectric particles) has no real effect on the sensing parameters of the metamaterial sensor, provided the dielectric particles fall on the active area (cap-gap) for sensing. In our LC resonant metamaterial sensor, A change in the resonant frequency of the metamaterial structure can be brought about by any foreign substance deposited in the capacitive gap

region, thereby changing the effective dielectric constant (ε_{eff}) and thus the capacitance, resulting in a redshift in the resonance frequency (ΔF) with respect to the pristine LC circuit in the array. Redshift in the resonance frequency can be brought by changing the permittivity or concentration of the dielectric material which will in turn change the ε_{eff}.

10: BSA deposition F2F MM with standard Si (725 μm)

We performed impedance spectroscopy experiments with 725 μm thick Si substrate and 10^{-3}% BSA concentration. We achieved a ΔF response of -33.29 GHz for the 725 μm thick Si F2F MM sensor in optimal coupling configuration (figure S9) compared to -163 GHz for the 200 μm thick Si F2F MM sensor (shown in figure 4b of the main paper). Therefore the ΔF response increased by nearly 5 times by thinning the substrate, which is exactly what we had proved in our previous work. Also, due to the increased thickness we can observe increased FP periodicity and reduced quality factor of the resonance, as we had proved in our previously published paper. Moreover, for the thinned 200 μm Si F2F MM sensor 10^{-3}% BSA gave a ΔF of -163 GHz, and 200 μm Si single MM sensor gave a ΔF of -70 GHz, with a 2.4 times improvement. Similarly, for the standard 725 μm Si F2F MM sensor 10^{-3}% BSA gave a ΔF of -33.29 GHz, and 725 μm Si single MM sensor gave a ΔF of -12 GHz (published in our previous work, reference number 19 of the main paper) with a comparable 2.7 times improvement.

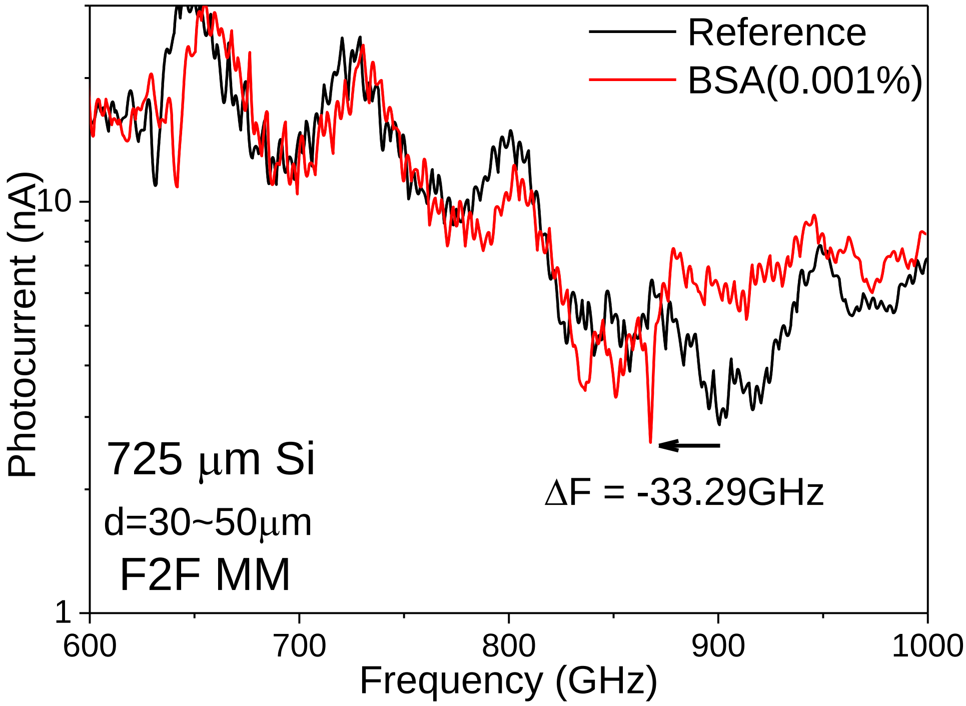

Figure S10. Experimental impedance spectroscopy measurements showing ΔF for BSA dielectric for F2F MM in the optimal coupling configuration fabricated on standard Si of 725 μm thickness.

11: BSA deposition on one metasurface experiment

In this experiment, we deposit BSA only on the front-metasurface and then, only on the back-metasurface. Figure S9 shows experimental impedance spectroscopy measurements showing ΔF when BSA dielectric is placed on the back metasurface only (blue line), front metasurface only (pink line) and both metasurfaces (red line) for F2F MM in optimal coupling configuration. In both the cases of BSA deposition on only one metasurface, we observe a $\Delta F_2 = -73\ GHz$, exactly analogous to the single MM on thinned 200 µm substrate, whereas $\Delta F_1 = -163\ GHz$ for BSA deposition on both metasurfaces.

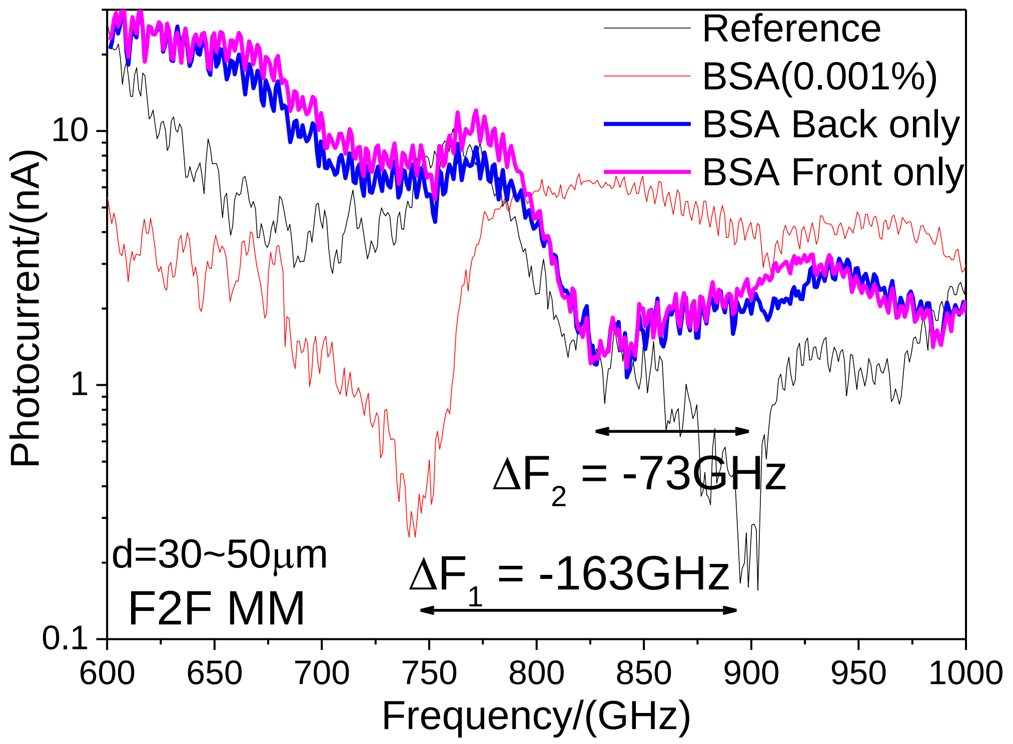

Figure S11. Experimental impedance spectroscopy measurements showing ΔF when BSA dielectric is placed on the back metasurface only (blue line), front metasurface only (pink line) and both metasurfaces (red line) for F2F MM in optimal coupling configuration.

12: Dielectric sensitivity calculation

In this work we calibrate the simulation FF with experiment dielectric concentration. After the experiments are conducted, we find the best FF suited to that concentration. Then we vary the FF linearly so that we get a similar effect in ΔF, as in experiments. Then the slope of FF vs ΔF (from simulations) and dielectric concentration vs ΔF (from experiment) is correlated, till the slope becomes identical. This will signify that the experimental sensitivity values will coincide with simulated sensitivity values

The sensitivity values are calculated in this work in the unit of GHz per refractive index unit (GHz/RIU) since this is a more widely used unit for sensitivity for comparison. We first estimate sensitivity can be estimated in terms of particle concentration with the linear fitting and slope calculation from the simulation results. The slope shown in figure 2(a) in the main paper is directly proportional to the sensitivity

in terms of particle concentration. Since ΔF is directly proportional to the effective change in refractive index (RI) due to presence of dielectric in the active region ($\Delta F \propto 1/n_{eff2} - 1/n_{eff1}$, n_{eff2} is the effective RI after adding the external dielectric and n_{eff1} is the effective RI before adding the external dielectric), sensitivity is then re-calculated in terms of refractive index unit (RIU). n_{eff2} is calculated using the formula, $n_{eff2} = \sqrt{\varepsilon_{eff} + \alpha N(\varepsilon_f - 1)}$ where ε_{eff} is the effective dielectric constant without the deposition of the dielectric, α is the coefficient which is associated with the surface fraction of the nano cubes or dielectrics, ε_f is the dielectric constant of one block of the cube, and N denotes the number of dielectric particles on the active area; and $n_{eff1} = \sqrt{\varepsilon_{eff}}$ [2–4]. The ΔF is then divided by the difference between n_{eff2} and n_{eff1}, to get the sensitivity in terms of RIU.

13: Experimental parameters summarization table

Table S1: Summary of the experimental ΔF for F2F MM in optimal coupling configuration with respect to various analytes and their concentrations

**Analyte Type**	**Analyte Concentration**	**ΔF (GHz)**
Nanoparticle (Salt)	-	-28
Nanoparticle (PbN)	-	-61.5
Nanoparticle (ZnO)	-	-68
BSA	0.001%	-90
	0.0001%	-110
	0.00001%	-140
	0.000001%	-163
Sugars	20 mg/Dl	-145
	30 mg/Dl	-150
	40 mg/Dl	-159
	50 mg/Dl	-168
	60 mg/Dl	-176
	70 mg/Dl	-185
	80 mg/Dl	-194
	90 mg/Dl	-202
	100 mg/Dl	-211
	110 mg/Dl	-222
	120 mg/Dl	-230
	130 mg/Dl	-239
	140 mg/Dl	-251

	150 mg/Dl	-259
**Sensitivity**	**2300 GHz/RIU**	

Table S1 provides a detailed information of experimental parameters such as analyte type, analyte concentrations, and ΔF for F2F MM in optimal coupling configuration.

14: Comparison with other complex sensitivity optimization strategies

Other advanced metamaterial architectures like the plasmonic nanostructure metamaterials need a higher degree of precision in the manufacturing process including expensive lithography techniques (like e-beam lithography). Plasmonic nanostructures and hybrid resonators also feature high sensitivity sensors and can be tailored for different sensing purposes or light manipulation. But making a THz sensor based on these principles will significantly increase complexity, cost and even scalability. We use simple Si substrate and one-level photolithography to print the metasurface design on the substrate, flowed by aluminum deposition and lift-off. We also follow the most cost effective automated mechanical polishing machines to thin down the substrate. The metamaterial sensor production steps we follow are completely CMOS compatible and therefore is extremely cost effective. This is the reason we made a comparison of our sensitivity parameters with other metamaterial genres or metamaterial architectures that only sense dielectrics and are operating in THz. In other words, our F2F MM sensor in optimal coupling configuration is an easy to fabricate CMOS compatible sensor with one of the highest sensitivities recorded till date.

References

[1] A. J. Deninger, A. Roggenbuck, S. Schindler, S. Preu, *J. Infrared, Millimeter, Terahertz Waves* **2015**, *36*, 269.

[2] R. Sengupta, H. Khand, G. Sarusi, *Sens. Bio-Sensing Res.* **2024**, *44*, 100639.

[3] S. J. Park, S. H. Cha, G. A. Shin, Y. H. Ahn, *Biomed. Opt. Express* **2017**, *8*, 3551.

[4] S. J. Park, J. T. Hong, S. J. Choi, H. S. Kim, W. K. Park, S. T. Han, J. Y. Park, S. Lee, D. S. Kim, Y. H. Ahn, *Sci. Rep.* **2014**, *4*, 4988.
